# Draft Genome Sequence of *Microbacterium* sp. Strain CH12i, Isolated from Shallow Groundwater in Cape Hallett, Antarctica

**DOI:** 10.1128/genomeA.00789-14

**Published:** 2014-08-14

**Authors:** Eloy R. Ferreras, Pieter De Maayer, Thulani P. Makhalanyane, Leandro D. Guerrero, Jackie M. Aislabie, Don A. Cowan

**Affiliations:** a Centre for Microbial Ecology and Genomics, Department of Genetics, University of Pretoria, Pretoria, South Africa; b Landcare Research, Hamilton, New Zealand

## Abstract

The Antarctic continent is largely covered by an expansive ice sheet, but it harbors diverse terrestrial and aquatic habitats in the coastal ice-free continental margins. Here we present the draft genome of *Microbacterium* sp. CH12i, which was isolated from hypersaline, alkaline, and nutrient-rich groundwater from Cape Hallett, northern Victoria Land, Antarctica.

## GENOME ANNOUNCEMENT

During the summer season, shallow groundwater may be present in the coastal areas of Antarctica. A psychrotolerant bacterium, *Microbacterium* CH12i, was isolated from shallow groundwater associated with ornithogenic sites at Seabee Hook, Cape Hallett, in northern Victoria Land, Antarctica. Groundwater at this site was alkaline and had high concentrations of salt and nutrients (1). Members of the genus *Microbacterium* (phylum, *Actinobacteria*; family, *Microbacteriaceae*), which comprises 84 validly published species (http://www.bacterio.net/microbacterium.html), are Gram-positive, aerobic, and heterotrophic bacteria that are widespread in nature, having been isolated from soil, water, plants, dairy products, insects, and humans (2). CH12i was grown on R2A agar and incubated at 15°C for up to a month (1). DNA was extracted using a combination of bead-beating and chemical lysis methods modified from Miller and colleagues (3). The CH12i genome was sequenced using an Ion Torrent PGM sequencer (318 chip) (Life Technologies) with 400-bp chemistry. After quality control filtering and trimming using in-house scripts, 2,268,858 reads were assembled with MIRA v. 4.0rc4 (4). The resulting contigs were subsequently merged and assembled using Gap5 (5), yielding 21 contigs with an average size of 159,750 nucleotides (nt) and a maximum length of 492,196 nt. The draft genome of *Microbacterium* sp. CH12i is approximately 3.35 megabases in size, with a mean G+C content of 63.84%. The genome was annotated using the Rapid Annotation using Subsystems Technology (RAST) server (6) and the NCBI Prokaryotic Genome Annotation Pipeline (http://www.ncbi.nlm.nih.gov/genome/annotation_prok), identifying 3,751 protein-coding genes and 51 RNAs, including 45 tRNAs, and 2 complete rRNA operons.

Analysis of the annotation output of RAST revealed that the CH12i genome codes for a number of proteins involved in resistance to antibiotics and toxic compounds, as well as a large number of stress-related proteins, including both heat-shock and cold-shock proteins, oxidative and osmotic stress proteins, and capsular polysaccharide biosynthesis proteins. The availability of several complete and draft genomes of temperate *Microbacterium* strains will allow comparative studies to elucidate factors underlying its ability to survive the harsh conditions to which *Microbacterium* sp. CH12i is exposed in its natural environment, including mechanisms to tolerate low temperatures and high salinity and alkalinity.

### Nucleotide sequence accession numbers.

This whole-genome shotgun project has been deposited in DDBJ/EMBL/GenBank under the accession number JHET00000000. The version described in this paper is version JHET01000000.
